# Time Trend Analysis of the Prevalence and Incidence of Diagnosed Asthma and Traditional Chinese Medicine Use among Adults in Taiwan from 2000 to 2011: A Population-Based Study

**DOI:** 10.1371/journal.pone.0140318

**Published:** 2015-10-20

**Authors:** Yi-Chun Ma, Cheng-Chieh Lin, Sing-Yu Yang, Hsuan-Ju Chen, Tsai-Chung Li, Jaung-Geng Lin

**Affiliations:** 1 Graduate Institute of Chinese Medicine, College of Chinese Medicine, China Medical University, Taichung, Taiwan; 2 Tai-An Hospital, Taichung, Taiwan; 3 Department of Family Medicine, China Medical University Hospital, Taichung, Taiwan; 4 School of Medicine, College of Medicine, China Medical University, Taichung, Taiwan; 5 Department of Medical Research, China Medical University Hospital, Taichung, Taiwan; 6 Graduate Institute of Biostatistics, China Medical University, Taichung, Taiwan; 7 Management Office for Health Data, China Medical University Hospital, Taichung, Taiwan; 8 Department of Healthcare Administration, College of Medical and Health Science, Asia University, Taichung, Taiwan; Universität Bochum, GERMANY

## Abstract

**Background:**

The aim of this study was to determine the annual trends of traditional Chinese medicine (TCM) use for prevalent and incident asthmatic adults in Taiwan from 2000 to 2011. The annual prevalence and incidence of asthma in adults among subgroups of sociodemographic factors were also investigated.

**Methods:**

A population-based study was conducted using a random sample with one million beneficiaries of all residents aged ≥18 years enrolled in the National Health Insurance program. Adults diagnosed with asthma were identified from the National Health Insurance Research Database. The annual prevalence and incidence of asthma in the adult population were estimated by using International Classification of Diseases, Ninth Revision, Clinical Modification diagnostic codes to identify relevant cases from 2000 to 2011.

**Results:**

The number of annual prevalent cases of diagnosed asthma increased from 56,885 in 2000 to 101,535 in 2011. The prevalence increased significantly on annual basis, whereas the incidence rate fluctuated over time. The prevalence of TCM use by adults with asthma decreased significantly (p<0.05), from 38.58% in 2000 to 29.26% in 2011. The number of annual incident cases of diagnosed asthma decreased from 3,896 in 2000 to 2,684 in 2011. TCM use rates in asthma incident adults decreased significantly (p<0.05), from 54.24% in 2000 to 38.19% in 2011.

**Conclusion:**

The prevalence of TCM utilization is high among adults with asthma in Taiwan. However, our study demonstrated a substantial decrease in the annual prevalence of TCM use by prevalent and incident asthmatic adults in Taiwan from 2000 to 2011. In addition, the prevalence of TCM use was higher among incident cases, compared with those with prevalent cases.

## Introduction

Asthma is a chronic inflammatory respiratory disease with partially or completely reversible airway obstruction. The most common symptoms are coughing, dyspnea, and chest tightness. Worldwide estimates of the prevalence of adult asthma vary widely, from 0.8% to 13.4%.[1] Ethnicity and demographic and environmental factors may contribute to these diverse variations. Asthma is a major chronic disease in Asia and the epidemiological burden of the disease has been investigated in children. Few studies have followed this line of study in Asian adults, particularly in ethnic Chinese people.

Asthma is associated with adverse outcomes, which could lead to inability to work, hospitalization, disability, and morbidity. The disease places a heavy burden on governments, health care systems, patients, and their families. In Taiwan, the severity of asthma increases after 18 years of age and the mortality is high in the elderly.[2] The health care costs for hospital outpatient visits, urgent visits and hospitalization in adults with asthma are above 2 times of those without asthma.[3] Thus, investigating the prevalence and incidence of asthma in adults is crucial. However, though numerous studies have examined the epidemiology of asthma among children in Taiwan,[4–8] studies of the epidemiology of asthma among adults are scant. Only three previous studies have investigated the prevalence of adult asthma in Taiwan, and only one has examined the incidence. Among these studies, one enrolled a population of all ages and used imprecise estimates of prevalence of adult asthma,[9] and the others were based on small local populations with limited nationwide representativeness or used a questionnaire design with limited diagnostic validity and recall bias.[10–12] More recent data based on large sample sizes is required to more accurately determine the prevalence and incidence of asthma among adults in Taiwan. Furthermore, some existing demographics factors must be investigated to refine these prevalence and incidence estimates. This study could assist in identifying potential risk factors and planning public health policy.

The current treatment suggested for asthma is aimed at controlling the disease, such as through an inhaled corticosteroid or the combined use of an inhaled corticosteroid and a long-acting β2 agonist or leukotriene receptor antagonist. Some patients worry about the adverse effects of these drugs and thus seek integrated or alternative treatments. Using traditional Chinese medicine (TCM) to treat asthma is common in Taiwan, with asthma-related visits accounting for approximately 3.5% of visits to TCM practitioners between 1996 and 2001.[13] Hence, using a comprehensive nationwide database to evaluate the time trends of TCM utilization in prevalent and incident asthmatic adults in Taiwan is necessary.

In Taiwan, the National Health Insurance (NHI) program provides universal health insurance. Implemented by the NHI Administration of the Ministry of Health and Welfare, the NHI program was initiated in March 1995 and now covers approximately 99% of the 23.74 million residents of Taiwan.[14] Besides, TCM health care is also covered by the NHI program in Taiwan and TCM is prescribed by licensed TCM physicians. The National Health Insurance Research Database (NHIRD), one of the largest insurance databases in the world, includes comprehensive information on beneficiaries’ demographic data, clinical visit dates, diagnostic codes, prescription details, expenditure amounts, and other data. NHIRD recorded the entire information of TCM prescriptions, which includes drug names, drug doses, and administration days. The database is publicly available and released by the NHI for research. This study used nationwide NHI claims data to determine the annual trends of TCM utilization for prevalent and incident asthmatic adults in Taiwan from 2000 to 2011. We also estimated the time trends in the prevalence and incidence of adult asthma and investigated the sociodemographic factors that may explain the changes in these prevalence and incidence estimates.

## Materials and Methods

### Data sources

The NHIRD contains claims data of one million beneficiaries randomly selected from all residents aged ≥18 years enrolled in the NHI program between 2000 and 2011. The International Classification of Disease, Ninth Revision, Clinical Modification (ICD-9-CM) was used to identify diseases. The study population for each specific year was defined. All data were anonymized upon inclusion in the NHIRD. Our study was exempted from institutional review board approval by the Public Health, Social and Behavioral Science Committee Research Ethics Committee of China Medical University Hospital.

### Incident and prevalent asthma diagnoses

Adults diagnosed with asthma between 2000 and 2011 were identified from claims data sets. If adults had at least three outpatient visits for asthma (ICD-9-CM code 493 or A code A323) or at least one inpatient visit for asthma diagnosis in one year, they were defined as an asthma prevalent case in that specific year. Incident cases were those in which patients had no asthma diagnoses in the claims data sets of the calendar year before their first asthma diagnosis. The NHIRD contained all health care claims data since 1996. Incident cases between January 1, 2000 and December 31, 2011 were ascertained. The date of the first outpatient visit or hospital admission that met the definition for asthma, whichever came first, was used as the incident event date. Patients remained classified as prevalent cases if they remained in the data set.

### TCM use for asthma and sociodemographic factors

TCM use, specifically for treating asthma, was estimated. Only TCM ambulatory care was analyzed in this study, because the NHI does not cover TCM prescriptions for inpatient care.

The sociodemographic factors studied included age, sex, insurance premium, and urbanization level of residence area. Age was categorized into five levels: 18–30, 30–40, 40–50, 50–60 and ≥60 years. Insurance premiums were determined according to monthly salary and categorized into four levels: NT$0–20,000, NT$20,000–40,000, NT$40,000–60,000, and ≥NT$60,000. The residential areas of the patients were classified into seven levels of urbanization, according to the method developed by Liu et al. [15]

### Statistical analysis

The annual prevalence and incidence rates of clinically diagnosed asthma in adults between 2000 and 2011 were estimated. The prevalence of TCM use among prevalent and incident asthma adults was also evaluated. The estimates of the annual prevalence rates according to sociodemographic categories were obtained by dividing the number of prevalent adults of asthma in a year by the number of adults enrolled in the NHI program in that year. The annual incidence rates were estimated according to sociodemographic categories by dividing the number of adults newly diagnosed with asthma in a year by the number of adults enrolled in the NHI program who did not have asthma at the beginning of that year.

The prevalence and incidence rates of adult asthma were adjusted using a direct standardization method that employs the sex- and age-specific rates of each year, and the sex and age distributions of the study population for the year 2000. Multivariate Poisson regression models were used to analyze the trends in prevalence and incidence over time while controlling for sex, insurance premium salary level, and urbanization level changes.

## Results

A total of 102,875 patients with asthma in the NHIRD between 2000 and 2011 were identified as prevalent diagnosed cases. The mean age of the prevalent diagnosed cases was 56.82 years, with a standard deviation (SD) of 20.78. The mean age at death for persons who were dead annually from the source population of prevalent cases was 68.2 years (SD: 20.0 years) in 2000 and 76.9 years (15.2 years) in 2011 and the corresponding values for persons who were dead annually from the source population of incident cases was 69.2 years (20.9 years) in 2000 and 77.8 years (14.3 years) in 2011. The source population from which the prevalence was estimated was 751,514 in 2000 and 880,902 in 2011.

**Fig 1:**
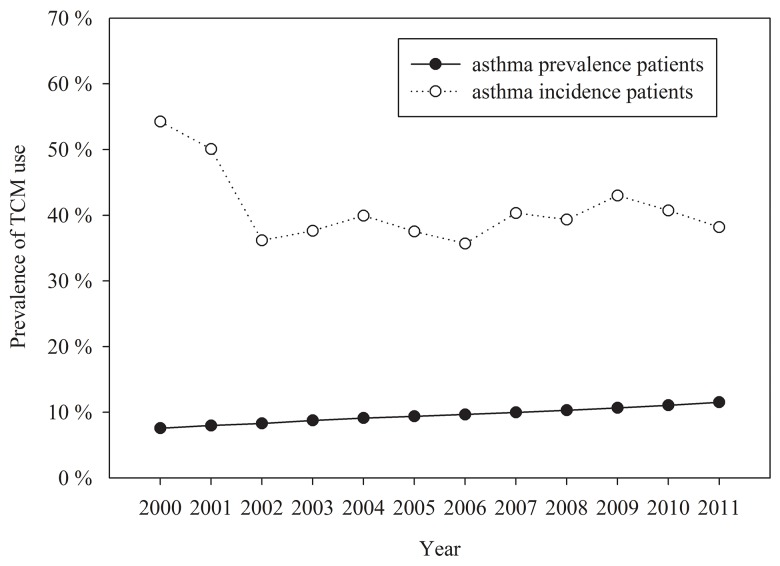
Time trends in the prevalence of TCM use among asthma prevalent and incident patients.

**Fig 2:**
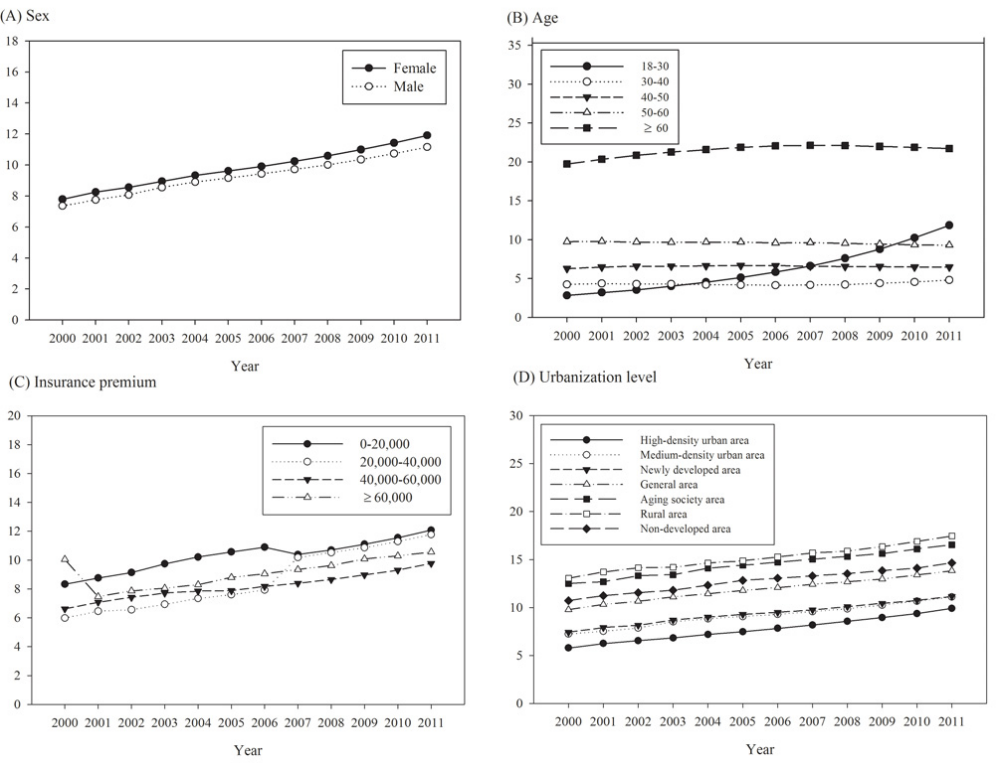
Time trends in the prevalence of asthma stratified by (A) sex (B) age (C) insurance premium (D) urbanization level.

The crude annual prevalence of diagnosed asthma increased 34.35% over the study period, from 7.57% of the adult population in 2000 to 11.53% in 2011 (Table 1). After direct standardization of the population in 2000, the annual standardized prevalence rates, which were slightly lower than the crude rates, increased over time. TCM use prevalence by asthma prevalent adults decreased significantly (p for trend <0.05), from 38.58% in 2000 to 29.26% in in 2011 (Fig 1). Higher annual asthma prevalence rates were observed in patients who were aged ≥60 years, female, had a low insurance premium salary level, or resided in a rural area or aging society. The annual prevalence rate increased dramatically in the group aged 18–30 years, from 2.84% in 2000 to 11.85% in 2011 (Fig 2).

**Table 1:** Prevalence and incidence of asthma in Taiwan.

	2000	2001	2002	2003	2004	2005	2006	2007	2008	2009	2010	2011
**Prevalence rates of asthma**												
**Prevalent cases**	56,885	60,593	64,266	67,709	70,195	75,081	78,767	82,551	86,650	91,096	95,952	101,535
**Age (year)**	55.1±18.7	55.4 ±19.0	55.9±19.4	56.6±19.6	57.1±19.9	57.2±20.4	57.4±20.8	57.6±21.2	57.6±21.8	57.5±22.3	57.3±22.9	57.1±23.4
**Age at death (year)**	68.2±20.0	72.9 ±14.0	74.7±12.0	75.2±12.1	76.6±11.4	75.8±12.9	75.3±13.5	76.4±13.4	76.5±14.3	76.7±13.5	76.7±15.0	76.9±15.2
**Total population**	751,514	758,012	773,155	773,213	770,002	799,830	814,823	827,555	841,577	854,044	867,049	880,902
**Prevalence rate (%)**	7.57	7.99	8.31	8.76	9.12	9.39	9.67	9.98	10.30	10.67	11.07	11.53
**Standardized prevalence rate (%)**	7.57	7.84	8.02	8.23	8.43	8.65	8.86	9.08	9.35	9.69	10.09	10.57
**Prevalence of TCM use (%)**	38.58	37.29	35.27	34.64	34.72	33.01	31.23	31.41	30.84	31.24	29.86	29.26
**Age**												
18–30	2.84	3.20	3.55	4.01	4.54	5.14	5.84	6.62	7.60	8.78	10.24	11.85
30–40	4.24	4.36	4.28	4.30	4.21	4.19	4.15	4.17	4.22	4.39	4.56	4.82
40–50	6.28	6.48	6.58	6.59	6.63	6.67	6.66	6.59	6.54	6.54	6.48	6.47
50–60	9.73	9.79	9.67	9.66	9.68	9.67	9.57	9.62	9.52	9.43	9.33	9.29
≥60	19.75	20.33	20.86	21.27	21.58	21.86	22.06	22.12	22.11	21.99	21.88	21.72
**Sex**												
Female	7.79	8.25	8.56	8.95	9.33	9.62	9.91	10.24	10.59	10.99	11.42	11.91
Male	7.36	7.75	8.08	8.56	8.90	9.16	9.43	9.72	10.01	10.35	10.73	11.16
**Insurance premium**												
0–20,000	8.33	8.76	9.14	9.74	10.21	10.56	10.89	10.38	10.69	11.10	11.55	12.07
20,000–40,000	5.98	6.45	6.56	6.93	7.34	7.60	7.93	10.18	10.52	10.86	11.29	11.76
40,000–60,000	6.62	7.08	7.42	7.71	7.85	7.88	8.17	8.39	8.65	8.97	9.30	9.76
≥60,000	10.05	7.47	7.87	8.05	8.29	8.80	9.04	9.35	9.62	10.08	10.29	10.56
**Urbanization level**												
High-density urban area	5.78	6.25	6.56	6.82	7.20	7.49	7.83	8.18	8.56	8.95	9.38	9.92
Medium-density urban area	7.24	7.52	7.87	8.52	8.82	9.07	9.28	9.58	9.86	10.26	10.67	11.09
Newly developed area	7.42	7.92	8.15	8.69	9.01	9.29	9.50	9.75	10.09	10.44	10.74	11.16
General area	9.77	10.33	10.65	11.1	11.44	11.77	12.08	12.42	12.68	12.97	13.40	13.83
Aging society area	12.51	12.69	13.32	13.42	14.10	14.41	14.72	15.04	15.33	15.62	16.11	16.53
Rural area	13.05	13.70	14.14	14.19	14.64	14.87	15.26	15.68	15.88	16.35	16.88	17.46
Non-developed area	10.73	11.23	11.55	11.80	12.33	12.83	13.05	13.29	13.54	13.84	14.11	14.66
**Incidence rates of asthma**												
**Incident cases**	3,896	3,541	2,792	2,379	2,564	2,569	2,263	2,318	2,163	2,211	2,230	2,684
**Age (year)**	51.7±18.4	52.2 ±18.5	55.6±18.5	55.5±18.1	54.1±18.6	55.3±18.2	55.4±18.6	54.7±18.3	55.9±18.1	54.2±18.0	54.8±18.3	54.5±18.1
**Age at death (year)**	69.2±20.9	73.2 ±16.0	77.4±10.7	76.6±11.5	76.0±14.9	75.8±14.7	77.8±12.2	76.4±12.6	76.8±14.8	76.2±11.8	77.6±14.9	77.8±14.3
**Population at risk**	698,525	686,061	697,407	692,443	685,211	695,714	719,768	729,719	739,134	749,231	757,324	766,327
**Incidence rate (%)**	0.56	0.52	0.40	0.34	0.37	0.37	0.31	0.32	0.29	0.30	0.29	0.35
**Standardized incidence rate (%)**	0.56	0.50	0.38	0.32	0.34	0.33	0.28	0.28	0.25	0.26	0.26	0.31
**Prevalence of TCM use (%)**	54.24	50.07	36.17	37.62	39.94	37.52	35.70	40.34	39.34	43.01	40.72	38.19
**Age**												
18–30	0.26	0.26	0.16	0.14	0.20	0.16	0.16	0.16	0.13	0.15	0.17	0.21
30–40	0.36	0.32	0.21	0.19	0.21	0.23	0.19	0.20	0.19	0.21	0.20	0.25
40–50	0.50	0.44	0.28	0.24	0.29	0.25	0.21	0.22	0.20	0.24	0.20	0.25
50–60	0.75	0.68	0.53	0.45	0.45	0.44	0.37	0.37	0.35	0.32	0.33	0.39
≥60	1.38	1.22	1.11	0.88	0.85	0.86	0.71	0.68	0.62	0.54	0.55	0.60
**Sex**												
Female	0.61	0.56	0.42	0.37	0.41	0.40	0.34	0.36	0.34	0.34	0.34	0.42
Male	0.51	0.48	0.38	0.31	0.34	0.34	0.29	0.27	0.25	0.25	0.25	0.29
**Insurance premium**												
0–20,000	0.60	0.54	0.44	0.38	0.41	0.40	0.34	0.28	0.24	0.26	0.24	0.30
20,000–40,000	0.49	0.47	0.32	0.28	0.32	0.32	0.28	0.35	0.34	0.33	0.35	0.39
40,000–60,000	0.49	0.53	0.36	0.30	0.32	0.36	0.29	0.32	0.27	0.26	0.26	0.32
≥60,000	0.69	0.44	0.34	0.34	0.31	0.33	0.29	0.32	0.28	0.31	0.25	0.37
**Urbanization level**												
High-density urban area	0.43	0.40	0.34	0.29	0.32	0.32	0.30	0.31	0.27	0.29	0.28	0.36
Medium-density urban area	0.53	0.54	0.40	0.34	0.37	0.36	0.29	0.31	0.29	0.30	0.29	0.34
Newly developed area	0.55	0.50	0.38	0.30	0.34	0.35	0.29	0.29	0.26	0.25	0.27	0.31
General area	0.79	0.65	0.47	0.45	0.44	0.44	0.36	0.34	0.32	0.32	0.33	0.37
Aging society area	0.72	0.67	0.63	0.41	0.70	0.45	0.40	0.35	0.42	0.32	0.41	0.47
Rural area	0.85	0.70	0.69	0.52	0.57	0.52	0.48	0.45	0.44	0.41	0.39	0.43
Non-developed area	0.80	0.71	0.50	0.48	0.51	0.55	0.35	0.39	0.37	0.32	0.26	0.35

**Fig 3:**
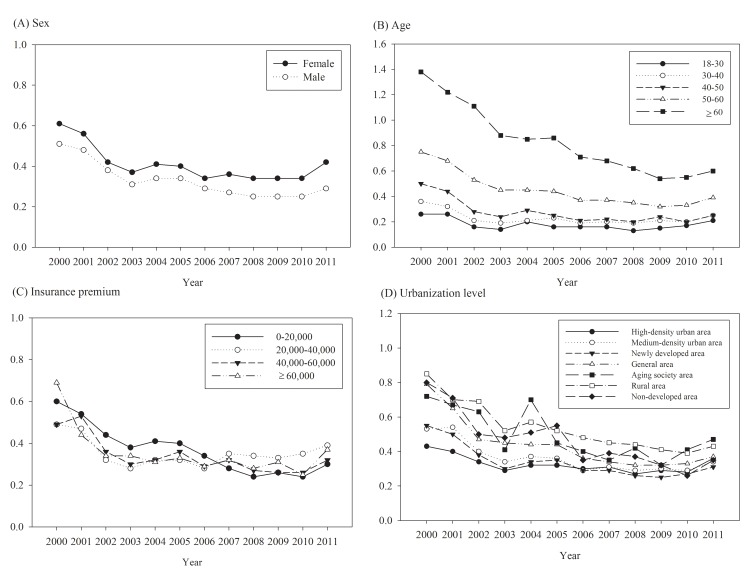
Time trends in the incidence of asthma stratified by (A) sex (B) age (C) insurance premium (D) urbanization level.

The number of annual incident asthma diagnoses decreased from 3,896 in 2000 to 2,684 in 2011 (Table 1). The mean age of incident patients was 54.49 years, with an SD of 18.31. The crude annual incidence fluctuated between 0.29% and 0.56% throughout the study duration. After direct standardization of the Taiwanese population in 2000, annual incidence rates decreased gradually and then slightly increased after 2009. The rate of TCM use by asthma-incident adult decreased from 54.24% in 2000 to 36.17% in 2002 and then fluctuated between 35.70% and 43.01% throughout the rest of the study duration (Fig 1). Higher annual asthma incidence rates were observed in patients who were aged ≥60 years, female, or resided in areas with low urbanization levels, such as an aging society and rural and undeveloped areas (Fig 3).

After multivariate adjustment, asthma prevalence was significantly associated with age (prevalence ratio [PR]: 1.31, 95% confidence interval [CI]: 1.27–1.35; 1.58, 1.53–1.63; 2.56, 2.48–2.64; and 4.69, 4.57–4.82 for the groups aged 30–40, 40–50, 50–60, and ≥60 years, respectively; Table 2). The prevalence increased annually with increasing relative risk (RR) as calendar year being treated as an ordinal variable (*P*<0.05). Prevalence was generally low among male patients (0.93, 0.92–0.93). The other major factors associated with prevalence rates were insurance premium salary level (0.99, 0.98–0.99 and 1.02, 1.01–1.02 for NT$40,000–60,000 and ≥NT$60,000, respectively) and urbanization level (1.15, 1.15–1.16; 1.19, 1.18–1.19; 1.30, 1.29–1.30; 1.28, 1.27–1.30; 1.42, 1.41–1.43; and 1.31, 1.30–1.32 for medium-density urban areas, newly developed areas, general areas, aging society, rural areas, and undeveloped areas, respectively). If we considered a trivial increase in the measure of association as a RR of less than 1.1 or greater than 0.91 [16] and a moderate or weak association as a RR of less than 3 or greater than 0.33 [17], the magnitude of association for age group of ≥60 years was strong, for sex and insurance premium level was trivial, and for calendar year, and urbanization level was weak.

**Table 2:** Multivariate-adjusted relative risk of annual prevalence of asthma rates for sex, age, time, insurance premium, and urbanization level.

Variable	Crude prevalence ratio(95% CI)	Multivariate-adjusted prevalence ratio (95% CI)
Poisson regression model- asthma prevalence		
**Calendar year**		
2000	1.00	1.00
2001	1.06 (1.05, 1.06)***	1.04 (1.03, 1.05)***
2002	1.10 (1.09, 1.11)***	1.07 (1.06, 1.08)***
2003	1.16 (1.15, 1.17)***	1.10 (1.09, 1.11)***
2004	1.20 (1.20, 1.21)***	1.13 (1.12, 1.14)***
2005	1.24 (1.23, 1.25)***	1.16 (1.15, 1.16)***
2006	1.28 (1.27, 1.29)***	1.18 (1.17, 1.19)***
2007	1.32 (1.31, 1.33)***	1.20 (1.19, 1.21)***
2008	1.36 (1.35, 1.37)***	1.22 (1.21, 1.23)***
2009	1.41 (1.40, 1.42)***	1.25 (1.24, 1.25)***
2010	1.46 (1.45, 1.47)***	1.27 (1.26, 1.28)***
2011	1.52 (1.51, 1.53)***	1.30 (1.29, 1.31)***
**Sex**		
Female	1.00	1.00
Male	0.95 (0.94, 0.95)***	0.93 (0.92, 0.93)***
**Age**		
18–30	1.00	1.00
30–40	0.73 (0.72, 0.73)***	0.72 (0.72, 0.73)***
40–50	1.10 (1.09, 1.11)***	1.09 (1.08, 1.09)***
50–60	1.61 (1.60, 1.61)***	1.57 (1.56, 1.57)***
≥60	3.62 (3.61, 3.64)***	3.44 (3.43, 3.46)***
**Insurance premium**		
0–20,000	1.00	1.00
20,000–40,000	0.92 (0.92, 0.93)***	1.00 (0.99, 1.00)
40,000–60,000	0.81 (0.81, 0.81)***	0.99 (0.98, 0.99)***
≥60,000	0.91 (0.91, 0.92)***	1.02 (1.01, 1.02)***
**Urbanization level**		
High-density urban area	1.00	1.00
Medium-density urban area	1.18 (1.18, 1.19)***	1.15 (1.15, 1.16)***
Newly developed area	1.20 (1.19, 1.20)***	1.19 (1.18, 1.19)***
General area	1.53 (1.52, 1.53)***	1.30 (1.29, 1.30)***
Aging society area	1.86 (1.85, 1.88)***	1.28 (1.27, 1.30)***
Rural area	1.95 (1.94, 1.96)***	1.42 (1.41, 1.43)***
Non-developed area	1.64 (1.63, 1.65)***	1.31 (1.30, 1.32)***

***: p<0.001.

Annual asthma incidence rates in adults fluctuated rather than followed a linear trend. Thus, considering time as an ordinal variable, we did not observe a significant annual increase in the incidence rate. The incidence rates among male adults were lower (RR: 0.80, 95% CI: 0.78–0.81). The other major factors associated with increasing incidence rates were age (1.31, 1.27–1.35; 1.58, 1.53–1.63; 2.56, 2.48–2.64; and 4.69, 4.57–4.82 for 30–40, 40–50, 50–60, and ≥60 years, respectively; Table 3), insurance premium salary level (1.12, 1.10–1.15 and 1.05, 1.03–1.08 for NT$20,000–40,000 and NT$40,000–60,000, respectively), and urbanization level (1.11, 1.08–1.13; 1.06, 1.03–1.08; 1.18, 1.15–1.21; 1.09, 1.03–1.15; 1.27, 1.22–1.32; and 1.19, 1.15–1.24 for the medium-density urban areas, newly developed areas, general areas, aging society, rural areas, and undeveloped areas, respectively). Among these significant factors associated with incidence, the magnitude of association for age group of ≥60 years was strong, for insurance premium level was trivial, and for calendar year, gender, and urbanization level was weak.

**Table 3:** Multivariate-adjusted relative risk of annual incidence rates of t asthma for sex, age, time, insurance premium, and urbanization level.

Variable	Crude relative risk(95% CI)	Multivariate-adjusted relative risk (95% CI)
Poisson regression model- asthma incidence		
**Calendar year**		
2000	1.00	1.00
2001	0.93 (0.90, 0.96)***	0.90 (0.88, 0.93) ***
2002	0.72 (0.69, 0.74)***	0.69 (0.67, 0.72) ***
2003	0.62 (0.59, 0.64)***	0.58 (0.56, 0.60) ***
2004	0.67 (0.65, 0.70)***	0.62 (0.59, 0.64) ***
2005	0.66 (0.64, 0.69)***	0.60 (0.58, 0.62) ***
2006	0.56 (0.54, 0.58)***	0.51 (0.49, 0.52) ***
2007	0.57 (0.55, 0.59) ***	0.49 (0.47, 0.51) ***
2008	0.52 (0.51, 0.54) ***	0.44 (0.43, 0.46) ***
2009	0.53 (0.51, 0.55) ***	0.44 (0.42, 0.46) ***
2010	0.53 (0.51, 0.55) ***	0.43 (0.42, 0.45) ***
2011	0.63 (0.61, 0.65) ***	0.50 (0.49, 0.52) ***
**Sex**		
Female	1.00	1.00
Male	0.81 (0.80, 0.82) ***	0.80 (0.78, 0.81) ***
**Age**		
18–30	1.00	1.00
30–40	1.25 (1.22, 1.29) ***	1.31 (1.27, 1.35) ***
40–50	1.48 (1.44, 1.53) ***	1.58 (1.53, 1.63) ***
50–60	2.34 (2.27, 2.41) ***	2.56 (2.48, 2.64) ***
≥60	4.34 (4.23, 4.46) ***	4.69 (4.57, 4.82) ***
**Insurance premium**		
0–20,000	1.00	1.00
20,000–40,000	0.91 (0.89, 0.92) ***	1.12 (1.10, 1.15) ***
40,000–60,000	0.87 (0.85, 0.89) ***	1.05 (1.03, 1.08) ***
≥60,000	0.85 (0.82, 0.88) ***	1.01 (0.97, 1.05)
**Urbanization level**		
High-density urban area	1.00	1.00
Medium-density urban area	1.11 (1.09, 1.13) ***	1.11 (1.08, 1.13) ***
Newly developed area	1.05 (1.03, 1.08) ***	1.06 (1.03, 1.08) ***
General area	1.35 (1.31, 1.38) ***	1.18 (1.15, 1.21) ***
Aging society area	1.51 (1.43, 1.59) ***	1.09 (1.03, 1.15) ***
Rural area	1.65 (1.58, 1.71) ***	1.27 (1.22, 1.32) ***
Non-developed area	1.42 (1.37, 1.48) ***	1.19 (1.15, 1.24) ***

***: p<0.001.

## Discussion

This is the first study to use nationwide NHI claims data to analyze annual trends of prevalence and incidence of adult asthma diagnoses and annual trends of TCM use among prevalent and incident asthma adults. Increases from 2000 to 2011 of 34.35% and 28.38% in the crude and age- and sex-standardized annual prevalence rates, respectively, were observed. This increasing trend in prevalence suggested that the disease burden may rise in the near future. Both the annual crude and standardized incidence rates fluctuated during the same period. The TCM use prevalence exhibited a decreasing trend and was much higher among incident than among prevalent asthma.

Regarding the prevalence of adult asthma in Taiwan, a nationwide study of adults and children from 2000 to 2007 reported an overall 8-year prevalence of 11.9% in the study population.[9] In addition, two previous studies were conducted in local populations through questionnaires. One of these studies reported adult bronchial asthma prevalence of 4.1% in Taipei City in 2004.[10] The other study reported asthma prevalence rates of 0.83% and 1.36% for men and women in Southern Taiwan in 2004.[11] Our estimates are closer to those of the nationwide study, and are much higher than those of the local population studies. Two possible explanations may account for these differences: geographic variation and measurement methodology. Regarding the incidence rate of asthma, the only previous related research was a local study that used questionnaires to estimate the incidence of adult asthma in Southern Taiwan in 2004.[12] The study reported incidence rates of 0.45, 0.83, 1.45, and 2.03 per 1,000 person-years in the groups aged 19–25, 26–30, 31–35, and 36–40 years, which are similar to our results. In this study, we used a different approach, namely retrospectively analyzing nationwide NHI claims data, to determine the annual prevalence and incidence of adult asthma diagnoses between 2000 and 2011.

Our study revealed significant increases in annual prevalence during 2000–2011. By contrast, annual incidence rates fluctuated throughout this period, and showed an overall decreasing trend. The asthma mortality rate has steadily decreased in Taiwan in the past decades, particularly among individuals aged ≥35 years.[18] Thus, improved asthma care with longer survival and more effective prevention methods may explain the increasing prevalence and decreasing incidence.

According to our estimates, the prevalence of adult asthma in Taiwan is relatively high. In the similar periods, our estimated prevalence rate was similar to those in Scotland (8.5%), the United States (7.21%-8.52%), and the United Arab Emirates (8%-12%); higher than those in Bangladesh (3.9%), China (0.8%-1.0%), Finland (3.5%), Hong Kong (5.8%), India (1.9%-2.9%), Iran (1.4%-6.1%), Japan (3.4%-4.2%), South Korea (2.4%-5.8%), Singapore (5.1%), and Thailand (2.9%); and lower than that in South Australia (12.2%-13.4%).[1,19,20] We observed a significant increasing trend in the prevalence of adult asthma in Taiwan, which has also been reported in South Australia, Finland, Hong Kong, Japan, South Korea, Scotland, and the United States.[1,19,20] As shown, Taiwan has one of the highest adult asthma prevalence rates in Asia. Despite the presence of environmental factors, such as high humidity, a subtropical climate, dense population, and air pollution [21] and rapid economic development, all of which are associated with increased risk of asthma, effective asthma care and decreasing mortality may explain the high and increasing prevalence.

Studies in other countries and regions have shown that the pooled rate of incidence asthma was approximately 4.6 per 1,000 person-years in women and 3.6 per 1,000 person-years in men and implied a trend of increasing incidence.[22] These rates were lower than our estimates for 2000–2001 but slighter higher than those for 2002–2011. According to our estimates, Taiwan has a relatively low asthma incidence rate. The incidence of asthma fluctuated during the study period and exhibited an overall decreasing trend, indicating the effectiveness of anti-air pollution efforts, including air pollution monitoring and warning systems and the regulation of air pollutant emissions by industry and traffic vehicles.

In our study, higher annual prevalence and incidence rates were observed in women, older people, low-income earners, and residents of rural areas. Recent epidemiologic studies worldwide have reported the prevalence of asthma in elderly populations as ranging from 4.5% to 12.7%.[23] Similarly, we observed high asthma prevalence and incidence rates in the group aged ≥60 years. The burden of asthma is greater on elderly people in mortality, hospitalization, and medical costs.[2] Thus, health promotion programs for asthma should be targeted at the elderly population.

Our study demonstrated that the prevalence of TCM use revealed a decreasing trend during the study period and was higher in incident (36.17–54.24) than in prevalent (29.26–38.59) adults with asthma. These findings may imply that adults with newly diagnosed asthma were more willing to receive or seek TCM as an alternative or supplemental treatment for asthma. Another possible explanation is that poor adherence to medications is a common problem among patients with chronic diseases and thus the prevalence of TCM use was lower among patients with longer asthma duration. A previous study showed that 85.7% of adults with asthma in Taiwan used TCM, which is a higher rate than that observed in the present study. Two possible reasons may explain the lower prevalence of TCM use in our study. First, the previous study estimated period prevalence, whereas we estimated annual prevalence. Second, we estimated TCM use prevalence specifically for treating asthma, whereas the previous study estimated TCM use prevalence among all patients with asthma, some of whom may have used TCM to treat conditions other than asthma. In addition, the previous study identified patients aged 20–29 years, residing in Northern and Central Taiwan, and with a monthly salary of NT$20,000–39,999 as being more likely to use TCM.[24] By contrast, the asthma-susceptible subgroups identified in this study, namely older patients, low-income earners, and residents of rural areas, were not common TCM users. This finding has critical implications for the planning of TCM promotion strategies, particularly those aimed at the target population. A major drop of prevalence of TCM use in incident adult with asthma from 50.07% in 2001 to 36.17% in 2002 was noted in this study. This can be explained by the introduction of inhaled steroid and long-acting β2 agonist combination Seretide or Symbicort in Taiwan since 2001, which changed the medical use condition.

The present study has several strengths. First, we provided valid and precise prevalence and incidence estimates because the nationwide sample we used is representative and large. Furthermore, this large sample size also facilitated subgroup stratified analysis, providing a more precise description of the prevalence and incidence rate estimates. Second, our study determined the annual prevalence and incidence trends of asthma in a Chinese adult population, literature about which was limited. Third, we investigated several sociodemographic factors that facilitate public health policy making and planning for health care utilization by asthma-susceptible groups. Fourth, we inspected the current TCM use by prevalent and incident asthmatic adults in Taiwan. Fifth, the NIH program in Taiwan covers nearly the entire population, thereby avoiding selection bias.

The limitations of this study warrant note. First, the study depended on claims data exclusively, which may result in the potential intentional or unintentional misclassification of diseases. To counter this, we included only patients who had at least three outpatient visits or at least one inpatient admission claim with an asthma diagnosis; thus, the prevalence and incidence may have been underestimated. Second, data on the severity of asthma, lung function, and laboratory tests were not available in the claims database; thus, combining this information with the prevalence and incidence data was beyond the scope of this study.

## Conclusion

This study showed that the annual prevalence of asthma among adults in Taiwan increased from 2000 to 2011, whereas the annual incidence fluctuated and showed an overall descending trend. TCM use by adults with asthma was common, but its annual trend had decreased. Appropriate health promotion programs are recommended, particularly for women, older patients, low-income earners, and residents of rural areas. Furthermore, more studies using large samples should be conducted to evaluate the cost-effectiveness of using TCM to treat asthma in adults.
